# Miscellanea

**Published:** 1855-06

**Authors:** 


### MISCELLANEA.

### SOLDIER LIFE IN INDIA.

During the discussion on the India Bill in 1853, the curious fact was elicited, that the Honourable East India Company's European regiments were absorbed and required to be renewed every eight years, while Her Majesty's regiments serving in India needed renewal every twelve years only. The causes of this difference have never yet been ascertained. In order, therefore, to throw some light on this subject, Dr. F. S. Arnott, surgeon of the Fusiliers, has drawn up a most elaborate report on the health and condition of the 1st Bombay European Regiment (Fusiliers), from the 1st of April 1845, to the 31st of March 1854.

This Report in its individual character cannot and does not attempt to settle the question referred to above; but it is an admirable history of the soldier's life in India, and as such we shall extract from it a few of the more important facts which it contains.

The regiment, in its eight years of service, has served within and without the Company's territories, within and beyond the tropics, and in climes ranging from 13 to 34 north latitude, and from 44 to 74 of east longitude; in high and low stations on the coast, and in arid plains. It has made long marches, and has been stationary; it has enjoyed a profusion of supplies, and has been limited to commissariat rations; it has had its ranks thinned by the fire of the enemy, and it has been exposed to great vicissitudes of temperature in crowded tents, with the thermometer at 114, and a few months afterwards at freezing point, with snow all around; it has been conveyed from place to place in small river steamers, and sometimes in large sea-going vessels; it has at times been located in small, confined, and comfortless pendals, and at other times in lofty, capacious, and commodious barracks. Dr. Arnott has seen sickness prevailing so little, that for two months the admissions to hospital did not reach 12 per cent. of the strength; and, on the other hand, he has seen in the same space of time the admissions into hospital exceed the entire strength of the regiment, at the rate of 120 per cent. With the strength exceeding 1000 men, he has known six consecutive months pass over with only one burial; and, again, he has witnessed funeral parties consign to their last resting place forty-eight of their comrades lost by disease in two consecutive days. He has seen the regiment decimated by disease in nine days, and reduced in the space of a year at the rate of 215 per 1000, which would have annihilated it in less than five years; and he has known the annual reduction as low as 47 per 1000,—a rate of decrement which would require twenty years for absorption. He has observed the discharged from service not to exceed one in twelve months, and he has lately seen them amount to twenty-two.

*Barrack Accommodation.*—Among the causes which lead to the deterioration of European troops serving in India, Dr. Arnott enumerates faulty accommodation in barracks, and more particularly in what are called the Patcheries, or places where the married soldiers live; also want of cleanliness, life on ship-board, and especially dress. On this last point we extract a few remarks.

*Dress.*—Although the dress of the soldier in India has improved, much more requires to be done. The Indian soldier in full dress must still put on the cloth coat of a northern climate, trowsers fit for a Siberian campaign, and must wear a hat that protects the head from neither heat nor cold. Efficiency is sacrificed to appearance, and soldiers serving in latitudes having a temperature of 50° above zero, and in countries with the mean annual of 50° above freezing point (that is nearly double the number of degrees of Fahrenheit above freezing point), are dressed alike. The stiff hard stock has happily been done away with, but the cumbrous shako, with its heavy metal ornaments, remains; and in the last Burmese campaign, regiments went into action in full dress, and lost as many lives by *coup de soleil* as by the fire of the enemy. The present coatee also prevents the full expansion of the chest, and is loaded with useless weighty ornaments. No soldier can march in the cloth trowsers, and they are not worn more than six times a year. The undress of the Indian soldier is suitable, but the accoutrements worn by the men when on duty are most mischievous, by confining the chest, and adding great weight, and causing extreme exhaustion. The soldier in India never carries the knapsack: it is borne by no back except that of the camel.

*Rations.*—The rations of the Indian soldier are very satisfactory: they now include vegetables and all necessaries; and should any doubt exist as to the qualities of the articles supplied, a committee of one medical and two military officers examine and report upon them.

*Water Supply and Malt Liquors.*—The water supply is generally good, except at Aden; and the advantages which have accrued to the soldier's health and morals by the substitution of sound malt liquor for ardent spirits, are already very perceptible in the diminution of crime, sickness, and mortality.

*Occupations and Amusements.*—In regard to occupations and amusements, the Indian soldier is the best cared for in the world; but he has still too much time on his hands, and idleness is the bane of his existence. Suitable employment is not always procurable: innocent games, therefore, and a taste for gardening should be more fully introduced.

*Furlough.*—Leave of absence home on furlough, which is now denied, would contribute much to the soldier's good.

*Diseases.*—In relation to the mortality arising from various diseases in the Fusilier regiment during the eight years of service, the report runs thus:—average strength of the regiment, 923; from cholera, 37½ per cent.; dysentery and diarrhœa, 22 per cent.

inflammation of the liver, $12\frac{1}{2}$ per cent.; fever, 8 per cent.; diseases of the chest, $8\frac{3}{4}$ per cent.; diseases of the head upwards of 5 per cent.; and of other diseases, including dropsy, peritonitis, small-pox, scurvy, and ulceration of the throat, upwards of 5 per cent. of total mortality.

*Changes in Indian Regiments.*—An interesting table, supplied by Dr. Arnott, gives an admirable idea of the internal changes to which an European regiment may be subjected in India during its eight years of service. From this table we learn, that the average strength of the regiment of Fusiliers for the eight years was 957, of whom 7 on an average were discharged, 2 deserted, 14 were pensioned, 11 were invalided, and $45\frac{1}{2}$ died per annum. The annual reduction of the regiment from all causes being 10·87 per cent., and from deaths 5·17 per cent.

*Healthy Seasons.*—At all the stations at which the regiment was located, January and February were the healthiest months of the year; next to these October.—(*Transactions of the Medical and Physical Society of Bombay*, 1853-54.)

---

### BIRTHS, DEATHS, AND MARRIAGES IN MASSACHUSETTS, CONNECTICUT, NEW JERSEY, AND KENTUCKY, U.S.

UNDER this title, the American *Journal of the Medical Sciences* publishes a most excellent paper, which throws much light on the social and sanitary conditions of the states above mentioned. The author's views on the subject of registration honestly merit the subjoined transcription.

"The work of registration is essentially connected with civilisation. The governments of all civilised nations desire to measure their power by counting their people, and calculating the amount of wealth, in order to show what amount of physical force they can present for offence or defence, and what means they have of sustaining themselves.

"In this view of the matter, the political economist sees that there is a great difference in the amount of wisdom, power, and happiness, that is contributed to the commonwealth, from the child in his forming period, and from the man in his full maturity; and the difference in the amount of good that has been contributed from the infant and the octogenarian is immense. A wise government, then, not only wants to know how many people exist, but how many are born, and how long they live. And besides all this, it wants to know how much that life is worth; what degree of fulness and vigour is, or may be, enjoyed through it.

"These matters are determined in great measure by the bills of mortality, which show at what periods life is terminated, and what are the causes of death. Those governments that count their people do well; those that determine their duration of life and the causes of death do better; and those do best of all which search out the obstacles that stand in the way of the most complete development of life, or impair its vigour, or prevent its maintenance, and endeavour to remove the cause of vital depression, which they may thus discover. This belongs to an advanced state of society, in which the ruling powers shall gather, digest, and diffuse, that knowledge which will lead the people to the fullest enjoyment of life."

### MASSACHUSETTS.

From the Registration Returns of the whole State of Massachusetts, there were reported :

| | Births. | Marriages. | Deaths. |
|---|---|---|---|
| 1849 | 25,773 | 6,936 | 20,423 |
| 1850 | 27,664 | 10,345 | 16,606 |
| 1851 | 28,681 | 11,966 | 18,934 |
| 1852 | 29,802 | 11,578 | 18,482 |

The average population of the whole State of Massachusetts for the four years 1849 to 1852 was :

| Males. | Females. | Total. | Area of Square Miles. | Inhabitants to one Mile. |
|---|---|---|---|---|
| 497,933 | 516,698 | 1,014,631 | 719.85 | 140.9 |

The average mortality for the same period was :

| Deaths per Year. | | | Rate of Mortality. | | | |
|---|---|---|---|---|---|---|
| Males. | Females. | Total. | Males. One in | Fem. One in | Male. Per cent. | Fem. Per cent. |
| 9,272 | 9,616 | 18,888 | 53.7 | 53.7 | 1.86 | 1.86 |

The average annual births and marriages during the same period was :

| Births. | | Marriages. | |
|---|---|---|---|
| Number. | Ratio to population. | Number. | Ratio to population. |
| 28,588 | one in 35.49 | 9,530 | one in 106.4 |

From another table supplied, it appears that the mortality in the great towns of Massachusetts is, as usual, much higher than in the rural districts. Thus to every 100 of both sexes dying in the country districts, not fewer than 155 die in the large towns.

It is further brought out that, in the city of Boston, the proportion of births and deaths amongst foreigners is much higher than amongst the American part of the population. Thus, while there were only 82 foreigners for every 100 American inhabitants, there were 121 deaths, 118 marriages, and 189 births among the former for every 100 among the latter. However, it should be remembered that these foreigners are generally among the poor, and belong to the labouring classes. They perform the hardest work, and are exposed to the greatest dangers of the elements, heat, cold, and wet. They, in the cities especially, live in crowded and unventilated houses and rooms. They are addicted to unhealthy habits of smoking tobacco and of drinking.

With regard to deaths from special diseases, the faulty diagnosis rendered in the registration returns makes this part of the subject of less value.

CONNECTICUT.

The reports for Connecticut are meagre and imperfect; however, the sum of them runs as follows:

| Years. | Births. | Marriages. | Deaths. |
|---|---|---|---|
| 1847-48 | 6,850 | 2,816 | 4,379 |
| 1848-49 | 7,238 | 2,920 | 5,000 |
| 1849-50 | 7,578 | 2,884 | 5,118 |
| 1850-51 | 8,360 | 2,995 | 4,712 |

NEW JERSEY.

The report from this state—also imperfect—runs thus for three years:

| Years. | Births. | Marriages. | Deaths. |
|---|---|---|---|
| 1851 | 11,861 | 2,859 | 5,089 |
| 1852 | 10,683 | 2,682 | 6,371 |
| 1853 | 12,107 | 3,388 | 5,651 |

KENTUCKY.

The report from this state is very satisfactory. It embraces an analysis of 25,906 births, 7,430 marriages, and 13,048 deaths.

*Conclusions.*—Taking into account all the reports, their facts and deficiencies, it appears with regard to the relative prevalence of the various causes of death, that diseases of the epidemic, endemic, and contagious classes, prevail in Massachusets, "in a proportion little more than half as large, and in New Jersey, in a proportion less than half as large as in Kentucky." The proportion of diseases of the nervous system is greater, both in Massachusetts and in Connecticut, than in Kentucky. There is an observable difference in the relative fatality of diseases of the lungs. The proportion of consumption is nearly equal in all the three eastern states, and more than twice as great in these as in the western state. But the proportion of every other pulmonary disease, except pleurisy, which was equal, was higher in Kentucky than in Massachusetts. Pneumonia, scarlatina, and croup, show a much higher ratio in the former than in the latter state. A similar difference is manifested in regard to asthma, bronchitis, hydrothorax, laryngitis, quinsy, and the unspecified diseases of the lungs. Deducting consumption, the result shows that of all other diseases of the respiratory organs 1,585 died in Kentucky, and 915 in Massachusetts, out of 10,000 deaths from all reported causes; and the whole burden of pulmonary diseases was relatively not so much greater in the eastern state, as the burden of the diseases of the digestive organs was less in the western state.

There was a marked excess in the ratio of dysentery, cholera, and fevers in Kentucky, over those in the other states. The ratio of deaths from old age was greater in Massachusetts and Connecticut than in Kentucky; but there was a larger class in which these deaths should take place. According to the census of 1850, the proportion of persons over 60 years old to the 10,000 of all ages was, in Massachusetts, 610; Connecticut, 773; and Kentucky, 357.

The warm season, in all of these states, was more fatal than the cold; there was the greatest difference in the months in Kentucky. Making corrections for the difference of length, there were, in that state, 150 per cent. more of deaths in August; and in Massachusetts 61 per cent. more in September than in February; and in Connecticut, 55 per cent more in July than in December.

In relation to age, it appears that the mortality in infancy was least in New Jersey, greater in Massachusetts, and much the greatest in Connecticut and Kentucky, in which it was nearly equal. The mortality amongst youths was about the same in Massachusetts and New Jersey, and was greater in these than in Connecticut and Kentucky, in which it was nearly equal. In the period of active manhood, the mortality is less in New Jersey and Kentucky than in the other states. The causes of these differences are not very obvious.

The report in no way shows satisfactorily what is the comparative mortality of the different states; the so called zymotic diseases, however, stand at a high figure; and consumption, as in this country, is the great disease.

The average longevity of the whole state of Massachusetts is 27.02 years. In Kentucky, amongst the white population, calculating from 9,631 deaths, the average duration of life was 20.55 years. Amongst the blacks, calculating from 3,367 deaths, the average longevity was only 17.59 years. The causes of this difference are not supplied, nor is there any similar return for the two remaining states.

---

### HORSEFLESH AS AN ARTICLE OF FOOD.

In the present year, M. Geoffroy St. Hilaire has devoted a part of his course in the Museum of Natural History in Paris to the consideration of applied zoology. Among the questions treated of, has been that of the consumption of horseflesh. Almost all our auxiliary animals at the same time furnish food; and this is easily understood; man, in multiplying these animals, creates at the same time a great mass of power and a large quantity of alimentary material; and he is led to avail himself of the latter when the former fails or becomes useless.

Why, then, does not the horse, a large animal, and the most extensively multiplied of our auxiliary quadrupeds, also furnish food? Like all herbivora, the horse produces an eatable flesh, rich in nitrogen, wholesome, and far from disagreeable to the taste.

Baron de Tott relates, that having been sent as an ambassador from the king of France to the Khan of Tartary, he was in the latter country entertained with an excellent meal of horseflesh. [The use of horseflesh as an article of food by the Tartars is a well-known fact.]

M. Huzard, a veterinary surgeon, relates that in 1789, the Parisians ate horseflesh during three months, and that the public health did not suffer in the least.

Baron Larrey, the celebrated military surgeon, says that horseflesh is very convenient as food for man; it seemed to him especially nutritious. He often saw it used, and with the greatest advantage, by the soldiers and the wounded of the French army. During the siege of Alexandria in Egypt, in order to overcome the repugnance of the soldiers to this article of diet, he killed his own horses and used them as food.

MM. Cadet, Parmentier, Pariset, and Parent-Duchatelet, have also reported favourably on the qualities of horseflesh.

Our repugnance to horseflesh arises simply from our long having ceased to use it. Anciently, both the horse and the ass were employed as articles of food. The use of horseflesh was at one time general among the inhabitants of the north and west of Europe. The reason for its disuse is thus given by M. Geoffroy St. Hilaire.

The worshippers of Odin used the horse in sacrifice. When the animal was sacrificed, the flesh was served up on the tables of the priests and of all classes of the population. The eating of horseflesh was thus connected with the rites of the Odin religion, and was a great obstacle to the establishment of Christianity among the people of the north: for, whenever a Scandinavian, even though converted, ate horseflesh, his mind reverted to the recollection of his former faith. Hence at an early period the popes prohibited the use of this article of food. In the eighth century, Pope Gregory III wrote to St. Boniface, archbishop of Mayence, to "abolish the custom by all possible means, and impose a proper penance on all eaters of horseflesh. They are unclean, and the act is execrable." His successor, Pope Zacharias, renewed the interdiction.

Now that the motive of the prohibition issued by the popes has disappeared for many years, the use of horseflesh is being gradually resumed; and it is remarkable, that it is first resumed by those who were the latest to abandon it. Denmark leads the way: in that country, horseflesh is sold publicly under the inspection of the government. For some years, Belgium has followed the example: and recently the Austrian government has authorised the public sale of this article of food.

It is to be hoped that France will not be the last country to throw off old prejudices. A wholesome, nutritious, economical article of food is lost in France by millions: and at the same time there exist millions of individuals insufficiently fed, and physically and morally deteriorated. The use of this article of food would regenerate them, and give to the state a class of robust and intelligent servants. If Ireland had been put in possession of this article of food, that country would perhaps not have offered the spectacle of one entire people torn by famine from their ancestral soil.

In conclusion, M. Geoffroy St. Hilaire observed that at first horseflesh must be regarded as food for the poor: it is in this character that its utility will be first shewn. The rich will use it if they please; and they ought to make use of it for the sake of example, and to prevent the poor from imagining that the use of horseflesh is one of the sad privileges of misery. (*Gazette Médicale de Paris,* 10th March, 1855.)

---

### SICKNESS AND MORTALITY IN FRANCE.

From a table constructed from observations in the hospitals of Paris for the year 1850, and republished in the *Assurance Magazine* for April 1855, it appears that masons, amongst males, present by far the highest figure as sufferers; next come the tailors, then house-painters, then bakers, then turners, then smelters; amongst these the *days of sickness* are longest in the masons, next longest in the tailors, next in the painters, next in the bakers, next in the turners, and next in the tanners. Of all these the *mortality* is highest in the tailors; the masons follow, and the other occupations in the order we have already given. Out of 6715 women attacked with sickness during the above period, not fewer than 4268, or nearly two-thirds, were confectioners.

In a remarkable work on the benevolent institutions of France, M. de Watteville, in 1851, stated the ratio of deaths in the hospitals of Paris to be 1 in 11, both for males and females. This was for the year 1847, which, from the dearness of provisions and the consequent misery in the poorer classes, must be considered exceptional. He estimates, also, the average duration of sick cases in the hospital as twenty-four days for males and twenty-five for females. The average mortality amongst female cases seems to be greater than amongst males, the former being 1 death for 11·94 patients, and the latter 1 in 13·92; but this may arise from females not having recourse to the aid of the hospital till the last extremity.

---

### CORONERS' INQUESTS IN ENGLAND AND WALES
### in the Years 1851, 1852, and 1853, and the Education of Jurors.

In 132 counties and boroughs of England and Wales, there were held in the year 1851, 16,847 coroners' inquests; in 1852, 17,656; and in 1853, 18,671; or 53,174 in the three years.

At each of these inquests there were on an average from twelve to fifteen jurors; supposing that there was an average of *fifteen* on each occasion, it would follow that 797,610 jurors were deputed to form an opinion as to the causes of deaths in 53,174 cases in which those causes were doubtful. It gives us no unimportant view of the frightful deficiency of education amongst the class of men who are summoned to sit on coroners' juries, most of whom are above the level of the labouring classes, to know that out of the 797,610 men thus summoned in the years above named, 34,433 at the lowest estimate, or 1 in 15, were unable to sign their own names.

### OBSTACLES TO VACCINATION IN INDIA.

On this subject, Mr. Atmaram Pandoorung, Graduate of the Grant Medical College, remarks, in a paper pubilshed in the *Transactions of the Medical and Physical Society of Bombay*, that amongst all classes of the natives of India, the distinction between vaccination and inoculation is not sufficiently understood, and that the want of success of inoculation is ascribed to vaccination. Instances of death from inoculation are not uncommon; and when one occurs, rumours are circulated calculated to injure the practice both of inoculation and of vaccination, but especially the latter. Again, there is a native belief amongst the ignorant that small-pox is sent by a certain deity in its mild or severe forms as a punishment for sin, and that it would be offering this deity an offence to interfere with his will.

Inoculation is practised in the Concan and Deccan under the name of the potter's process, which consists in scarifying the flesh freely and inserting the dried crusts removed from the eruption of small-pox. The practice of vaccination is called the Government process; and the writer believes that the natives believe in its efficacy, but that they exaggerate failures, and are not fully impressed with its importance.

Absence of information on the subject is therefore the chief cause of the want of success of vaccination in India. But there are other secondary causes; one of which is peculiar. The middle classes of the Hindoos are accustomed to expend large sums of money in celebrating the day upon which their children are either inoculated or vaccinated, and they are deterred from the practice in many instances on account of the expense implied. For extending vaccination, Mr. Pandoorung (whose opinion as a native ought to have great weight) deprecates coercion. He thinks that the employment of qualified physicians as vaccinators, and the circulation of tracts on vaccination in the vernacular language, would lead soonest to success.

---

### BRITISH SPIRITS EXPORTED TO THE COLONIE AND FOREIGN COUNTRIES.

In the years 1853 and 1854 there were exported from this country 1,076,619 gallons of *British and Irish spirits* to the British colonies and possessions. To foreign countries during the same period there were exported 431,857 gallons, making an aggregate export to all parts, of 1,508,476 gallons. Of the spirits imported to the colonies, 884,603 were sent to the Australian settlements only. To the foreign countries the main importation was to America, 343,641 gallons having been sent to the United States.

---

### SPIRITS CONSUMED IN SCOTLAND.

By a parliamentary return, bearing date March the 8th, 1855, it appears that in the year 1850, not less than 289,246 proof gallons of spirits, including *rum*, and all other *foreign and colonial spirits*, were cleared for home consumption in Scotland; 260,200 in 1851; 265,469 in 1852; 260,998 in 1853; 255,658 in 1854; and 29,230 in the months of January and February in 1855. In addition, there were cleared for home consumption, of *British spirits*, in 1850, 7,122,987; in 1851, 6,830,710; in 1852, 7,172,015; in 1853, 6,534,648; in 1854, 6,553,239; and in the months of January and February, 1855, 801,769. Thus taking the sum total we find, that in Scotland alone, from the 1st of January 1850, to the 1st of March 1855, there were consumed 36,376,169 proof gallons of alcoholic spirits.

To estimate the amount of immorality, disease, and misery which must needs have been dispensed by this distribution of fluids, which, as we all know, are absolutely unnecessary as articles of existence, would be impossible. But the great fact is before us as regards consumption, and the great evil may in some degree be comprehended as regards effects. Against such a wholesale system of suicide by poison, the science and art of medicine, great as they are, stand in insignificant array. We talk of the days of slow poisoning as events of the past, and as though they went out with the death of Cardinal de Bonzy. But what tale do the statements made above tell, as to slow poisoning at this present hour?

---

### FREQUENT RAILWAY TRAVELLING AS AN EXCITING CAUSE OF BRAIN DISEASES.

A peculiar combination of symptoms has of late been observed as a frequent result of railway travelling. Although this class of cases has recently attracted attention in some professional circles, it has not received any public notice. The matter is one of much importance, and it is very desirable that those who have had opportunities of observation should record their experience. Week by week the practice is becoming more common for gentlemen who reside in the country, at a distance from their place of business, to avail themselves of the railway as the means of daily transit. If it be true, therefore, as is beginning to be believed, that a few hours of train travelling daily may result in the production of disagreeable head symptoms, it is time that the public were put in possession of the fact.—(*Medical Times and Gazette*, June 9.)

[These remarks deserve great public attention; but it would be well to know, as a first step, whether stokers, engine-drivers, and guardsmen are subjected more than others to head affections. Into this subject we intend to inquire.—*Editor.*]

